# Effects of Biofilm Nano-Composite Drugs OMVs-MSN-5-FU on Cervical Lymph Node Metastases From Oral Squamous Cell Carcinoma

**DOI:** 10.3389/fonc.2022.881910

**Published:** 2022-04-19

**Authors:** Jian Huang, Zhiyuan Wu, Junwu Xu

**Affiliations:** Department of Oral and Maxillofacial Surgery, Fujian Provincial Hospital, Fuzhou, China

**Keywords:** outer membrane vesicle, mesoporous silica nanoparticle, oral squamous carcinoma, lymph node metastasis, drug release rate

## Abstract

This work was developed to the effects of biofilm composite nano-drug delivery system (OMVs-MSN-5-FU) on lymph node metastasis from oral squamous cell carcinoma. Mesoporous silica nanoparticles loaded with 5-FU (MSN-5-FU) were prepared first. Subsequently, the outer membrane vesicles (OMV) of Escherichia coli were collected to wrap MSN-5-FU, and then OMVs-MSN-5-FU was prepared. It was then immersed in artificial gastric juice and artificial intestinal juice to explore the drug release rate. Next, the effects of different concentrations of the nano-drug delivery systems on the proliferation activity of oral squamous carcinoma cell line KOSC-2 cl3-43 were analyzed. Tumor-bearing nude mice models were prepared by injecting human tongue squamous cell carcinoma cells Tca8113 into BALB/c-nu nude mice. They were injected with the OMVs-MSN-5-FU nano drug carrier system, and peri-carcinoma tissue and cervical lymph node tissue were harvested to observe morphological changes by Hematoxylin – eosin (HE) staining. The scanning electron microscope (SEM) results showed that all MSN, MSN-5-FU, OMV, and OMV-MSN-5-FU were spherical and uniformly distributed, with particle sizes of about 60nm, 80nm, 90nm, and 140nm, respectively. Among them, OMV had a directional core-shell structure. The cumulative drug release rates of artificial gastric juice in 48 hours were 61.2 ± 2.3% and 26.5 ± 3.1%, respectively. The 48 hours cumulative drug release rates of artificial intestinal juice were 70.5 ± 6.3% and 32.1 ± 3.8%, respectively. The cumulative release of MSN-5-FU was always higher than OMV-MSN-5-FU. The cumulative release of MSN-5-FU was always higher than OMV-MSN-5-FU. After injection of OMVS-MSN-5-FU, the number of cancer cells was significantly reduced and cervical lymph node metastasis was significantly controlled. HE staining results showed that OMVS-MSN-5-FU injection reduced the number of stained cells. Dense lymphocytes were clearly observed in the cortex of neck lymphocytes. The OMVs-MSN-5-FU drug delivery system can slow down the drug release rate, significantly inhibit the proliferation activity of oral squamous cancer cells, and control the metastasis of cancer cells to cervical lymph nodes.

## Introduction

Squamous cell carcinoma is a common malignant tumor of the head and neck, accounting for approximately 80% of all head and neck tumors, classified into oral cancer, laryngeal cancer, and nasopharyngeal cancer, etc. (1). Lymph node metastasis is an important cause of poor treatment effects and even death in cancer patients (2). Due to the special physiological anatomy of the oral maxillofacial region, most patients with squamous cancer are prone to neck lymph node metastasis, which seriously affects the prognosis (3). Data show that approximately 14% to 40% of patients with oral squamous cell carcinoma have cervical lymph node metastasis (4). Therefore, diagnosis of the cervical lymph node metastasis of oral squamous cell carcinoma is important (5). 5-Fluorouracil (5-FU) is a pyrimidine fluoride. It can inhibit the activity of thymine nucleotide synthase and is a commonly used anti-metabolism and anti-tumor drug. 5-FU has been widely used in the treatment of colorectal cancer, head and neck squamous cell carcinoma, and liver cancer (6–8). However, 5-FU can cause serious systemic side effects, such as mucositis and diarrhea, which is attributable to too little accumulation of 5-FU drugs in tumor tissue (9). Therefore, increasing the accumulation of 5-FU in the target area can enhance the efficacy of the drug and reduce the side effects.

Nowadays, nano-drug delivery systems play an important role in the pharmaceutical research and application, and nano-drug delivery systems prepared by encapsulating drugs in natural or synthetic polymer compounds can improve the therapeutic effects (10). By molding drugs into various nanostructures, they can be made more bioavailable and therapeutic. Polymer nanocarriers, solid lipid nanoparticles, nanostructured lipid carriers, nanoemulsions, nanodiamonds, vesicle-based drug carriers, metal-based nanoparticles, and nano-vaccines all have positive application effects as intelligent substitutes for drug delivery in the central nervous system (11). Nevertheless, the nano-preparation is only enriched in the liver or spleen, without a long-term circulation, and thus it can’t reach the targeted organs or tissue. To enhance the therapeutic efficiency of the nano-drug delivery system, to modify the cell membrane on the outer layer can significantly increase the drug loading. Additionally, the cell membrane has good biocompatibility, which also improves the stability of the nano-particles. Gram-negative bacteria outer membrane vesicles (OMV) are spherical biofilms, which are closed entities originating from endophytic cells (12). It can regulate the host’s immune response and participates a variety of biological and pathophysiological processes. Zhang et al. (13) improved the radiosensitivity of extranodal nasal NK/T cell lymphoma by combining radiotherapy with nano-drug delivery system, overcame the multi-drug resistance of chemotherapy drugs, and provided a new idea for the further development and optimization of treatment regimen for extranodal nasal NK/T cell lymphoma.

In this study, a biofilm composite nano drug delivery system (OMVs-MSN-5-FU) was prepared and immersed in artificial gastric juice and artificial intestinal juice to explore the drug release rate, and the effect of OMVS-MSN-5-FU on cervical lymph node metastasis was investigated. This study may provide a theoretical basis for the therapeutic effect of oral squamous cell carcinoma.

## Materials and Methods

### Laboratory Reagents

5-FU (Beijing Bailingwei Technology Co., LTD.); Cetyl trimethyl ammonium bromide (Jining Sanshi Biotechnology Co., LTD.); Ethyl orthosilicate (Tianjin Kermel Chemical Reagent Co., LTD.); (Tianjin Guangfu Fine Chemical Research Institute); Dimethyl sulfoxide (Jiangsu Haolong Chemical Co., LTD.); and Phosphate buffer salt solution (PBS, Hyclone Corporation, USA) were utilized. All other reagents were domestic analytical pure reagents.

### Preparation of Mesoporous Silica Carrier

**Figure 1:**
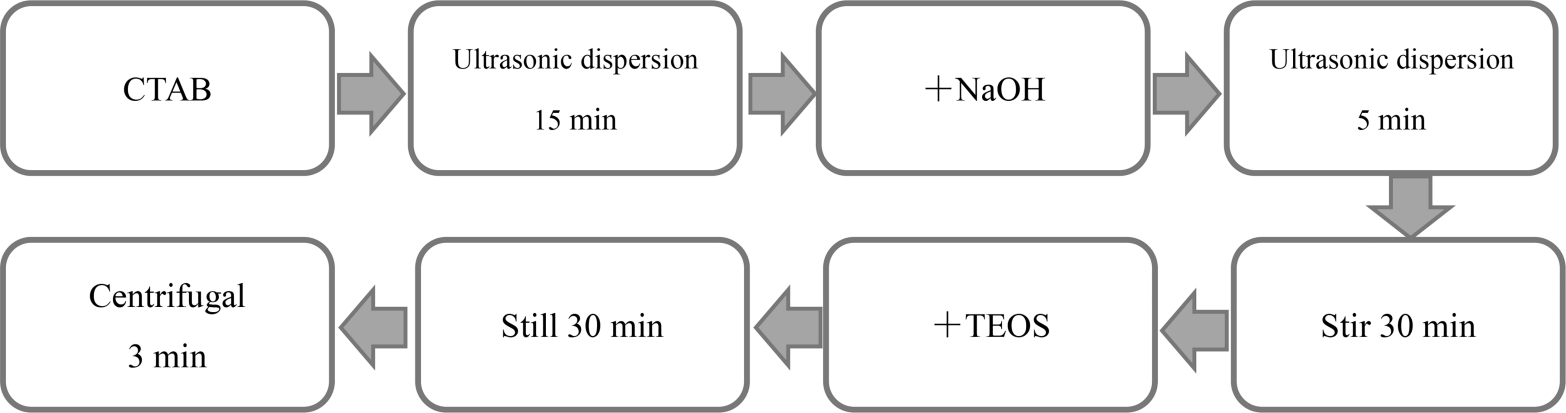
Flow chart of preparation of mesoporous silica nanoparticles.

The synthesis steps of mesoporous silica carrier were shown in **Figure 1**. 0.535g cetyltrimethylammonium bromide (CTAB) was dissolved in 240mL sterilized ultrapure water, and ultrasonic dispersion was performed for 15min. Then, 1.25mL of 2mol/L NaOH solution was added, followed by ultrasonic dispersion for 5min. The liquid was stirred continuously at 80°C for 30 minutes. Ethyl orthosilicate (TEOS) was added dropwise every 4s, totaling 5mL. After 2-hour reaction, the liquid was left at room temperature for 30min. The lower layer solution was taken for centrifugation at 10,000rpm for 3min to obtain the precipitate. The mesoporous silica nanoparticles (MSN) were then immersed in ethanol solution, followed by ultrasonic dispersion and centrifugation at 10,000 rpm for 3 minutes. The above steps were repeated three times. Next, the sample was transferred in a vacuum drying oven and dried overnight to obtain the purified MSN sample.

**Figure 2:**
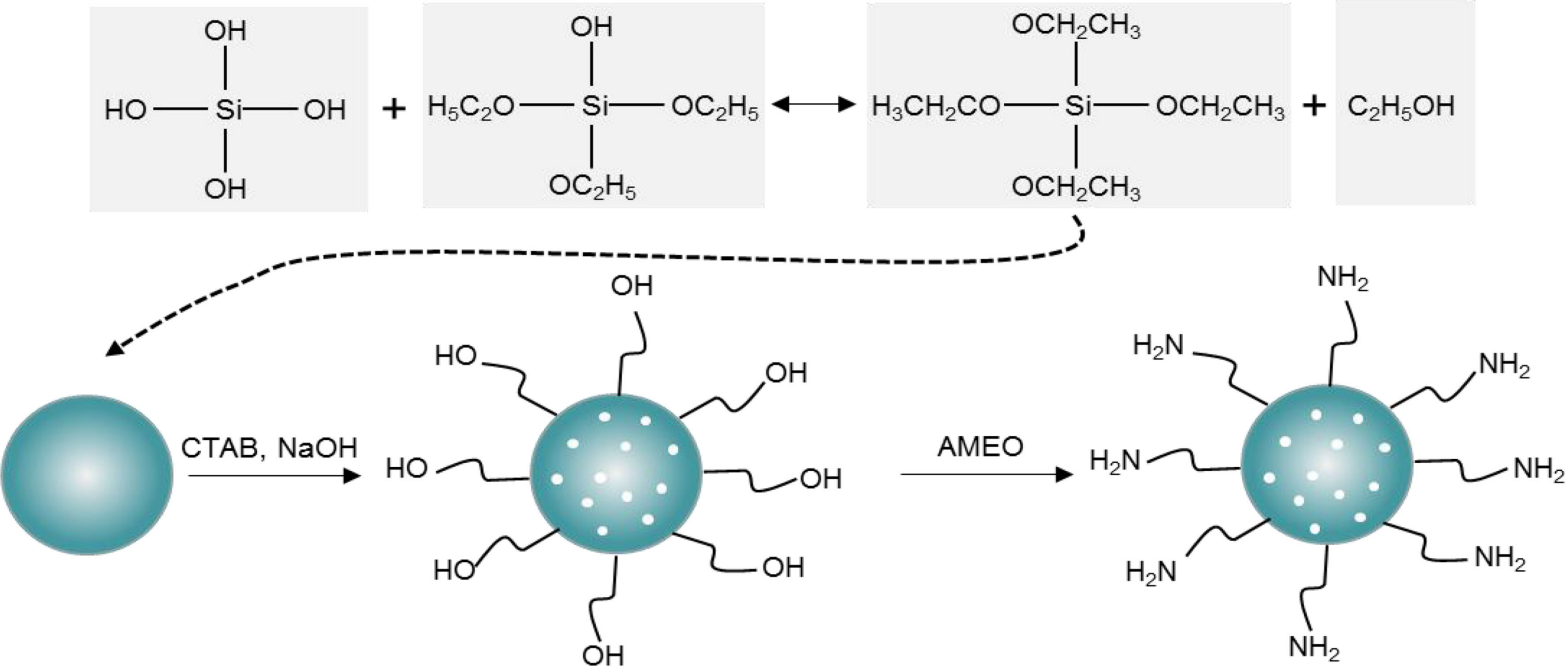
MSN surface amine process.

3-aminopropyltriethoxysilane (AMEO) was mixed with MSN at a ratio of 2:3, and an appropriate amount of toluene was added, followed by reflux under nitrogen protection for 12h at 110°C. Then, the solution was centrifuged at 10,000rpm for 3 mins, and the precipitate was washed with ethanol solution. The above steps were repeated three times. Finally, the surface amination treatment of MSN was carried out under vacuum drying conditions. The synthesis steps were shown in **Figure 2**.

### Preparation of MSN-5-FU

MSN-5-FU nanoparticles were prepared by reverse phase microemulsification. 7.5 mL cyclohexane, 1.6 mL hexanol, and 1.8 mL Triton-100 (surfactant) were mixed evenly, and 5-Fu solution was added to form an inverting microemulsion. Magnetic stirring was performed at room temperature for 5 min. 150 μL ethyl orthosilicate and 100 μL 25% ammonia were added to the mixture, and the reaction was stirred continuously at room temperature for 24 h. 2 mL acetone was added for demulsification and centrifugation for 20 min. The products were collected and dispersed with ethanol and water respectively, followed by centrifugation to remove unreacted 5-Fu and solvent. The final nanoparticles were vacuum-dried.

### Preparation of OMVs-MSN-5-FU

Luria-Bertani solid medium was used to cultivate Escherichia coli. After culturing at 37°C for 36 hours, a single colony was inoculated in Luria-Bertani liquid medium and continued to be cultured for 24 hours. 2mL of bacterial solution was inoculated in blank Luria-Bertani liquid medium, set as the blank control group. The culture was terminated when the OD600 was 1. Then, centrifugation was performed at 12,000 rpm for 20 min at 4°C. The supernatant was then passed through the 0.45μm disposable filter. The filtrate was transferred to 20mL ultrafiltration centrifuge tube, followed by centrifugation at 8000rpm at 4°C for 15min. The centrifugal liquid was concentrated to 1.5mL, and PBS was used for resuspension to obtain OMVs.

High-pressure nitrogen was used to extrude the OMVs to uniform their particle sizes. The above steps were repeated 3 times. OMVs and MSN-5-FU were mixed at a ratio of 1:5, and then extruded 6 times. The extruded effluent was centrifuged at 8000 rpm at 4°C for 20 minutes, and the supernatant was discarded. The bottom precipitate was OMVs-MSN-5-FU.

### Characterization Test

a. Transmission electron microscope (Talos F200X S/TEM, Beijing Opton Optical Technology Co., LTD.) and scanning electron microscope (LSM 900, Beijing Precise Instrument Co., LTD.) were used to observe the morphology of nanoparticles.
b. The Malvern Zeta particle size analyzer was used to measure the Zeta potential of nanoparticles, the measurement temperature was set to 25°C, and the equilibration time was 2 minutes.

### *In Vitro* Release Test of OMVs-MSN-5-FU Drug Delivery System

The release amount of OMVs-MSN-5-FU drug delivery system was determined through the immersion test of artificial gastric juice and artificial intestinal juice. The first step was to prepare artificial gastric juice and artificial intestinal juice. Preparation of artificial gastric juice: 1mol/mL dilute hydrochloric acid with pH=1.5 was mixed with 0.001g/mL pepsin. The mixture was then passed through 0.2μm disposable sterile filter membrane for filter sterilization. Preparation of artificial intestinal juice: 6.8g KH2PO_4_ was dissolved in 500mL sterile ultrapure water. With the pH of the solution set to 6.8, 0.01g/mL trypsin was then added. Next, the mixture was passed through a 0.2μm disposable sterile filter membrane for filter sterilization. Subsequently, 3mg of MSN-5-FU drug delivery system was added to 20mL of artificial gastric juice and 3mg OMVs-MSN-5-FU drug delivery system was added to another 20mL of artificial gastric juice, followed by shaking at 135rpm at 37°C. The artificial intestinal juice was treated in the same way. 2mL release solution was taken after 0.5h, 1.0h, 2.0h, 4.0h, 8.0h, 12.0h, 24.0h, 48.0h, and 72.0h of shaking, respectively, followed by centrifugation at 2000rpm for 5min at room temperature. Then, the supernatant was taken to determine the content of 5-FU.

### *In Vitro* Cytotoxicity Test of OMVs-MSN-5-FU Drug Delivery System

KOSC-2 cl3-43 cells were inoculated in a 96-well plate. 0, 0.1, 0.25, 0.5, 1, 2.5, 5, and 10μmol/L 5-FU, MSN-5-FU, and OMVs-MSN-5-FU were inoculated, respectively. At 24h, 48h, and 72h of culturing, 10μL of MTT solution was added to each well. Then, 100μL of dimethylsulfoxide solution was added to each well, followed by shaking for 10 minutes. Finally, the absorbance was measured at a wavelength of 570nm. Cell inhibition rate was calculated. The calculation method of cell inhibition rate is shown in equation (1), where *RI* was the cell inhibition rate, *AS* was the absorbance value of the experimental well, *AK* was the absorbance value of the blank well, and *AY* was the absorbance value of the negative well.


(1) $$RI=1-\frac{AS-AK}{AY-AK}\times 100\%$$

### Preparation of Tumor-Bearing Animal Models

The clean-grade BALB/c-nu nude mice were used, aged about 5 weeks old, weighing 16-21g, regardless of the gender. They were provided by the XXX animal laboratory. Animal License No.:SCXK(Hebei)2019-0027. In XXX animal laboratory, the day and night cycle was 12 hours at 22~26°C and 45~50% humidity. After three days of adaptive breeding, they were used in formal experiments.

Human tongue squamous cell carcinoma cells Tca8113 were cultured first, and PBS was used to prepare a single cell suspension at a concentration of 1×10^7^ during subculture. 10% chloral hydrate solution was intraperitoneally injected to anesthetize BALB/c-nu nude mice, and 0.2mL of Tca8113 cell suspension was injected **into the buccal mucosa**. The growth status of the tumor was observed regularly.

When the diameter of the tumor was approximately 0.5 cm, BALB/c-nu nude mice were anesthetized by intraperitoneal injection of 10% chloral hydrate solution, and 100μg/mL OMVs-MSN-5-FU suspension was injected into the buccal mucosa around the tumor in the oral cavity.

### Detection of Naa10 and Lymphocyte Subtypes in Peripheral Blood

Blood was drawn from the tail vein of the tumor-bearing animal model, and enzyme-linked immunosorbent assay was used to detect the level of Naa10 in the peripheral blood. The serum sample was transferred in an enzyme-labeled plate, and incubated at 37°C for 2 hours. After the supernatant was discarded, 100μL of test reagent A was added to each well. After 1 hour, the supernatant was discarded and the cells were washed with sterile ultrapure water 3 times. Then, 100μL of test reagent B was added to each well. After 1 hour, 90μL of enzyme-labeled reagent was added to each well, and 50μL of stop solution was added after incubation for 20min. Finally, the absorbance was measured using a microplate reader at 450nm.

Flow cytometry was used to detect the changes of T lymphocyte subsets (CD3^+^, CD^4+^ and CD^8+^), B lymphocytes (CD^19+^) and NK cells (CD^56+^) in peripheral blood.

### Observation of Tissue Sections

Three days after the injection of OMVs-MSN-5-FU suspension, the animal model was sacrificed by cervical dislocation, and the tissue around the carcinoma and ipsilateral cervical lymph nodes were harvested. The tissue was washed with PBS, and then put in the embedding box. Then, 70%, 90%, 95%, 95%, 100%, 100%, and 100% ethanol solution was used in turn to dehydrate the tissue, followed by immersion in xylene solution for 20 minutes. This step was repeated 3 times. The treated tissue was embedded in paraffin solution to made paraffin tissue sections with a thickness of 3μm. After being dried, the slide was immersed in xylene solution for 10 minutes, twice. Next, 100%, 100%, 95%, 95%, 80% ethanol solution and distilled water were used in turn for tissue rehydration. Subsequently, hematoxylin and eosin staining solutions were used to stain the tissue, followed by immersion in xylene. Finally, the slide was sealed using neutral gum, and visualized under an optical microscope.

### Statistical Analysis

SPSS19.0 was used to process the data. The data were all expressed by the mean ± standard deviation (*x*(-) ± s).One-way analysis of variance was used for statistical analysis of the differences between multiple groups, and *P<*0.05 was the threshold for significance.

## Results

### Transmission Electron Microscopy and Scanning Electron Microscopy Images of the Nano-Drug Delivery Systems

**Figure 3:**
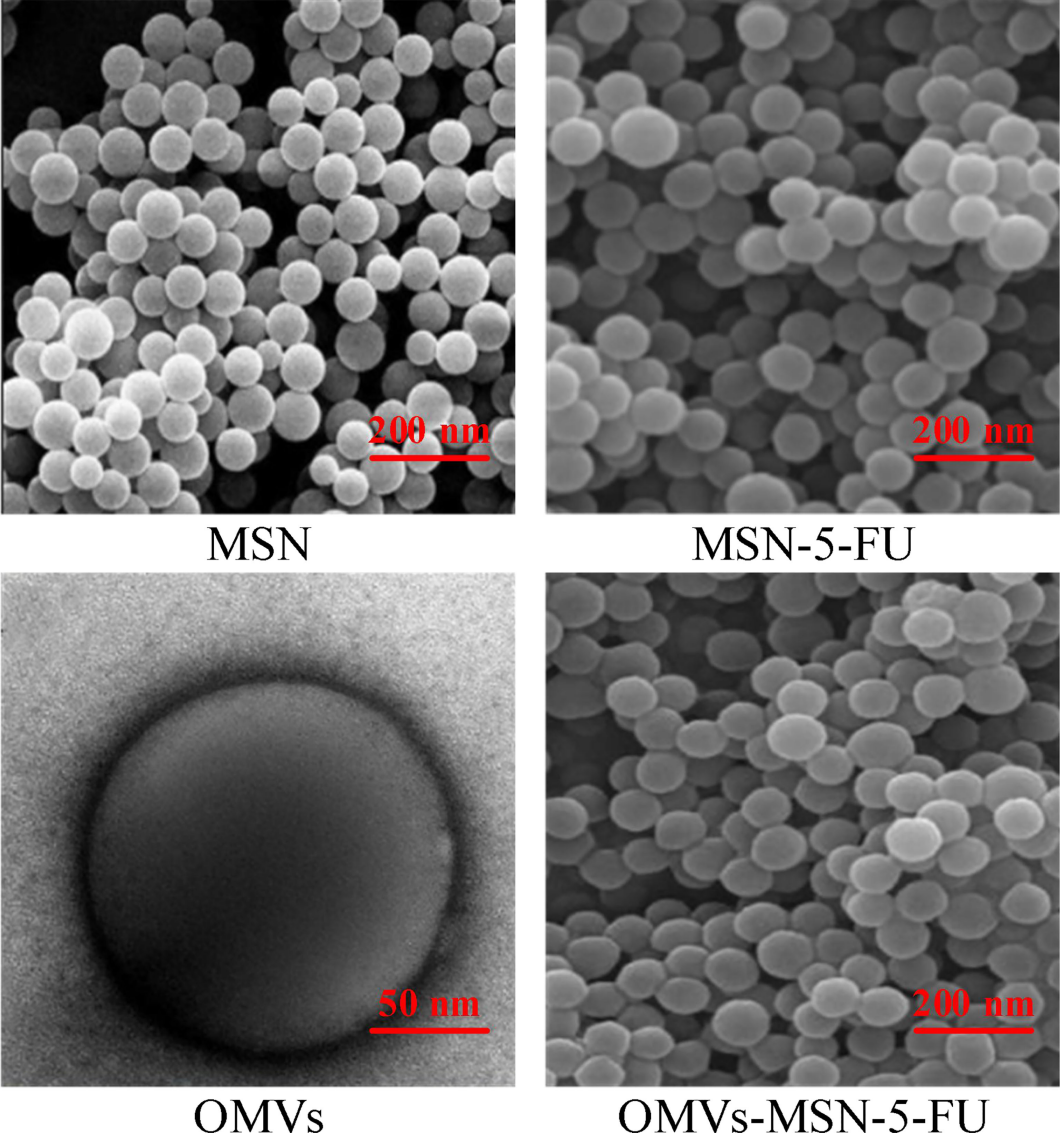
Scanning electron microscope images.

**Figure 4:**
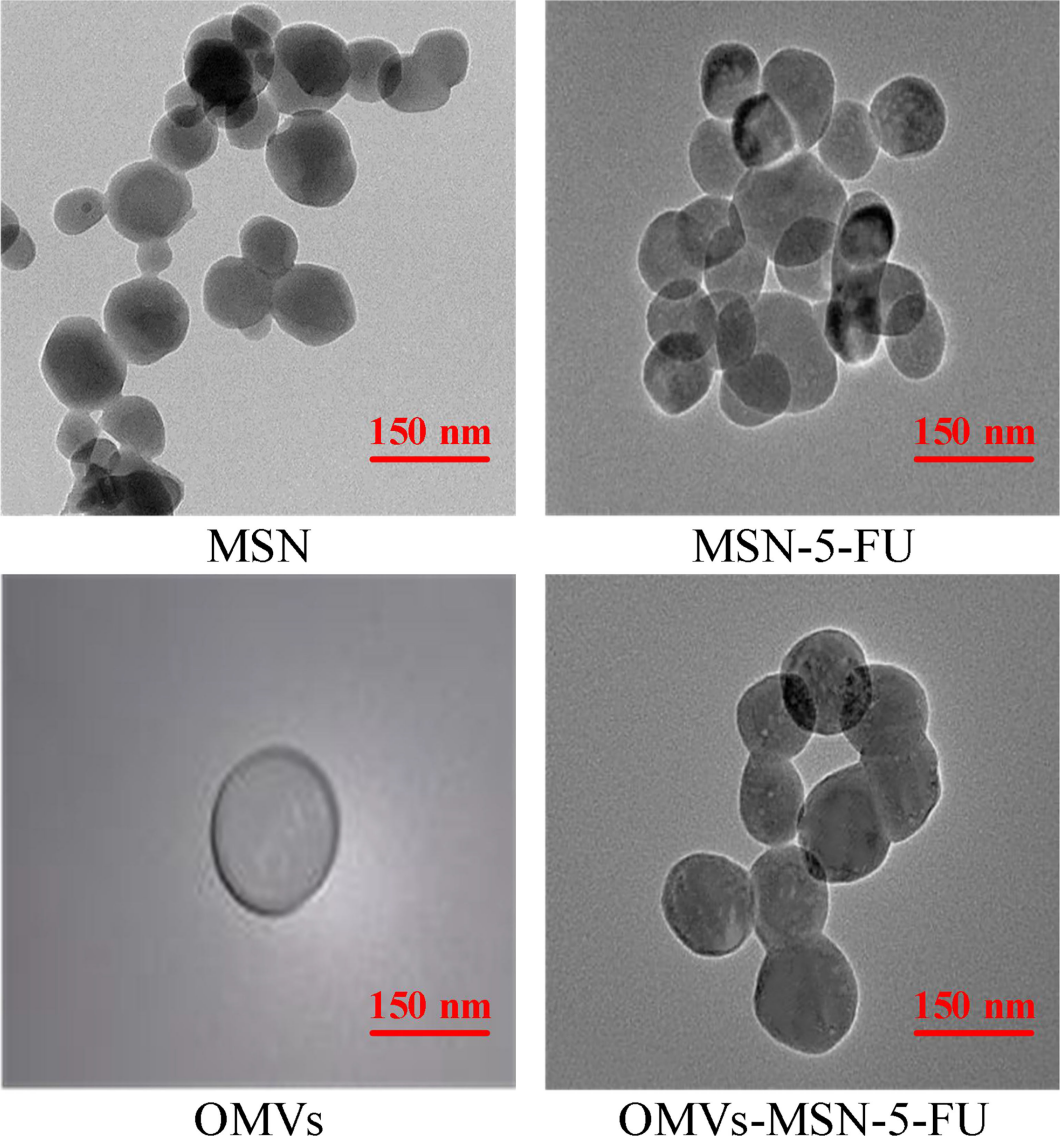
Transmission electron microscope images.

The morphology of MSN, MSN-5-FU, OMVs, and OMVs-MSN-5-FU was visualized under scanning electron microscope and transmission electron microscope. It was found that, all MSN, MSN-5-FU, OMVs, and OMVs-MSN-5-FU were spherical in shape, with uniform distribution. The particle diameters were approximately 60nm, 80nm, 90nm, and 140nm, respectively. Among them, OMVs had an oriented core-shell structure. The scanning electron microscope and transmission electron microscope results were shown in **Figures 3**, **4**.

### Zeta Potential Measurement Results

**Figure 5:**
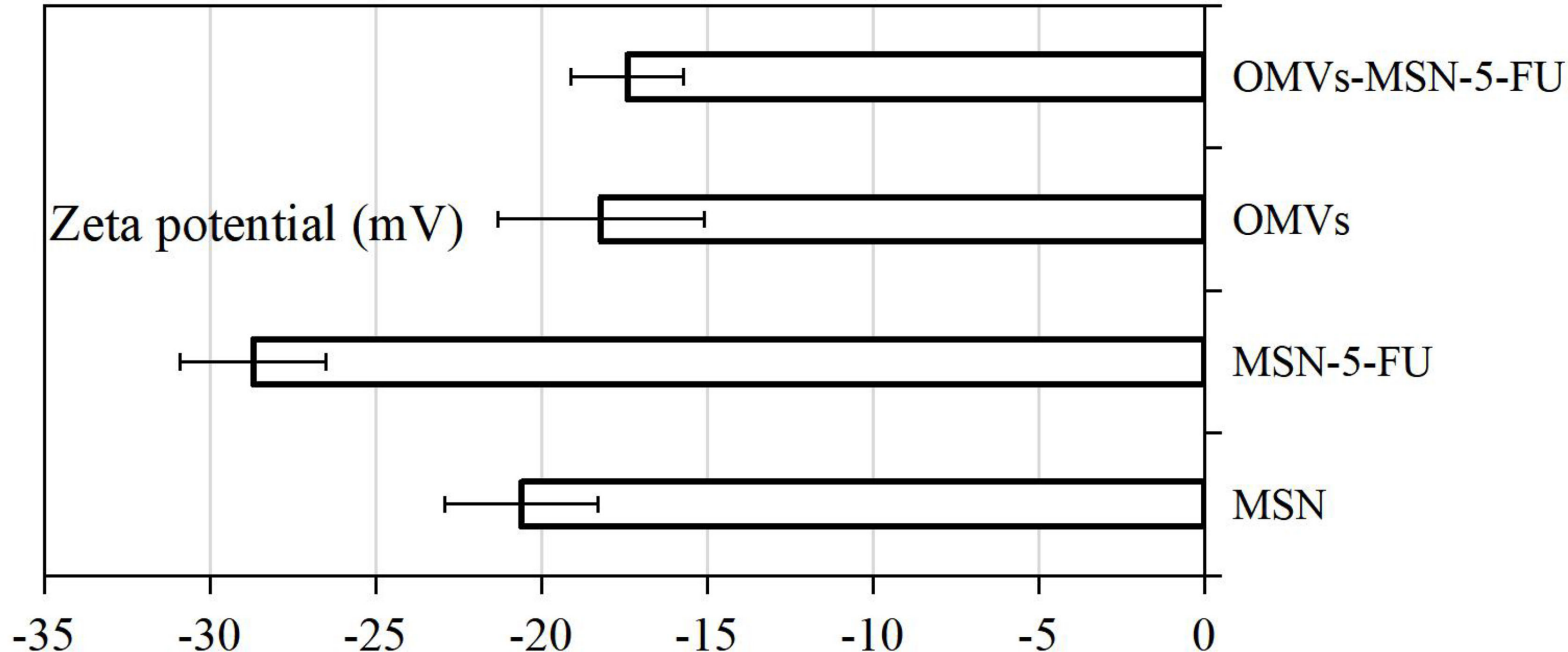
Average Zeta potential plot.

The average Zeta potentials of MSN, MSN-5-FU, OMVs, and OMVs-MSN-5-FU were -20.6 ± 2.3mV, -28.7 ± 2.2mV, -18.2 ± 3.1mV, and -17.4 ± 1.7mV, respectively. The average zeta potential of OMVs was close to that of OMVs-MSN-5-FU. The results of Zeta potential detection were shown in **Figure 5**.

### *In Vitro* Release of the Nano-Drug Delivery Systems

**Figure 6:**
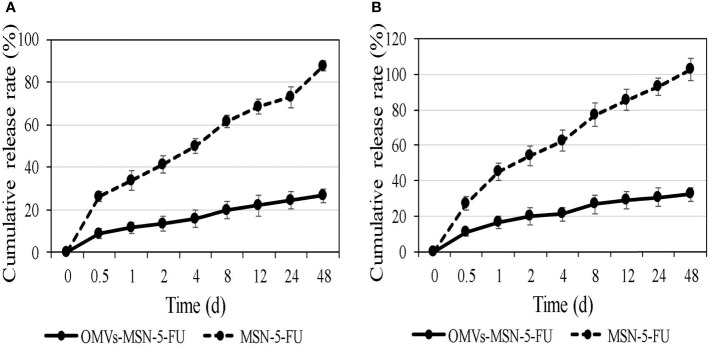
*In vitro* cumulative release rate curve of the nano-drug delivery systems. **(A)** was the cumulative release curve in artificial gastric juice; **(B)** was the cumulative release curve in artificial intestinal juice.

The *in vitro* release rate of MSN-5-FU and OMVs-MSN-5-FU drug delivery systems was analyzed. It was noted that, the cumulative release rate of MSN-5-FU and OMVs-MSN-5-FU drug delivery system gradually increased in artificial gastric juice and artificial intestinal juice. In addition, the 48-hour cumulative drug release rate in artificial gastric juice was 61.2 ± 2.3% and 26.5 ± 3.1%, respectively, and the 48-hour cumulative drug release rate in artificial intestinal juice was 70.5 ± 6.3% and 32.1 ± 3.8%, respectively. The cumulative release of MSN-5-FU was always higher than that of OMVs-MSN-5-FU. The cumulative release rate results were shown in **Figure 6**.

### Cytotoxicity of the Nano-Drug Delivery Systems

**Figure 7:**
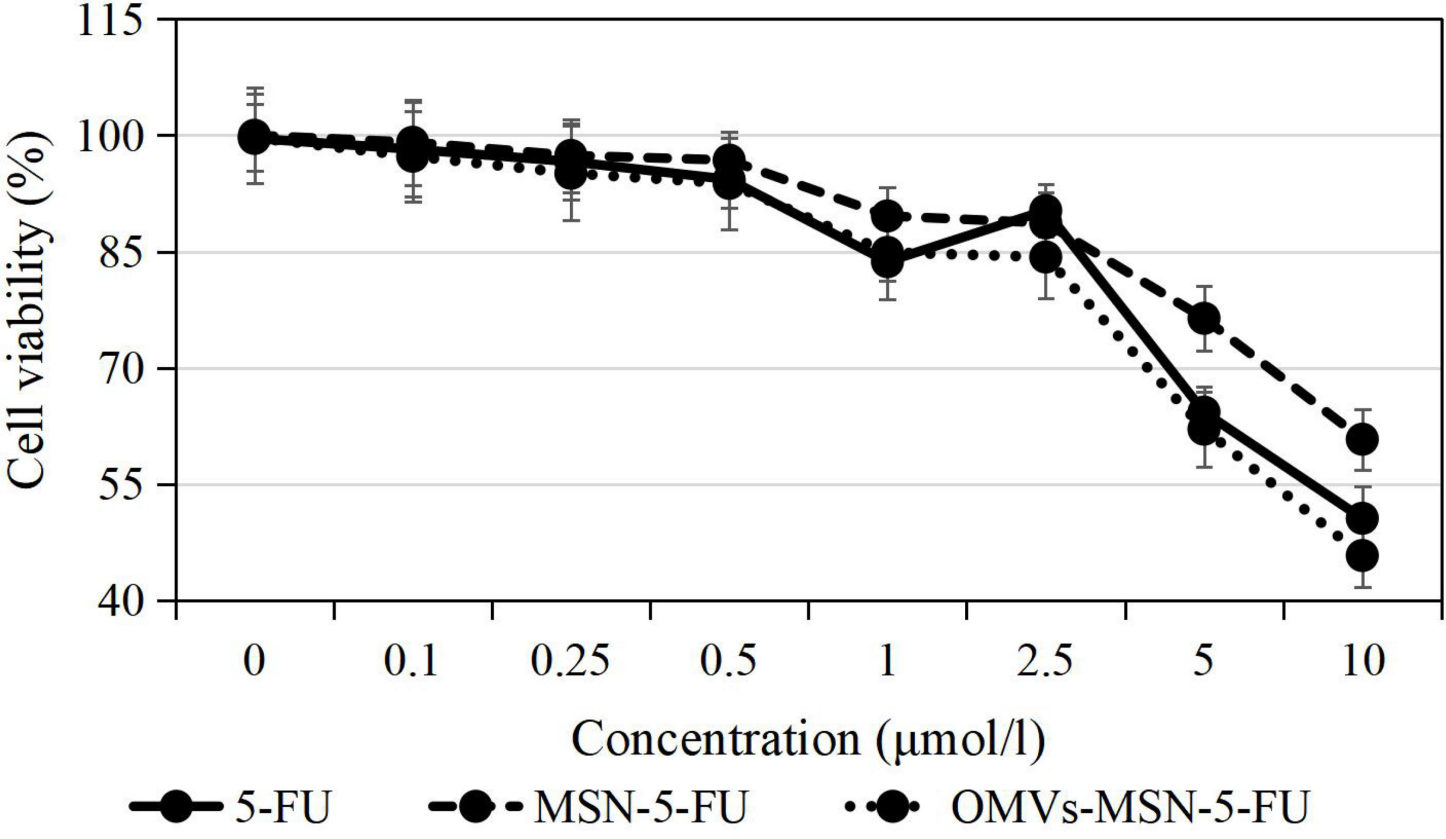
The inhibitory effects of free drug and the nano-drug delivery systems on cell proliferation.

The results of cell viability detected by MTT showed that, with the increase of the concentration of 5-FU, MSN-5-FU and OMVs-MSN-5-FU drug delivery systems, the proliferation activity of KOSC-2 cl3-43 cells showed a gradually decreasing trend. At the same time, under the same dosage, the inhibitory rate of OMVs-MSN-5-FU drug delivery system on the proliferation activity of KOSC-2 cl3-43 cells was higher than that of 5-FU and MSN-5-FU. The specific results were shown in **Figure 7**.

### Identification of Tumor-Bearing Animal Models

**Figure 8:**
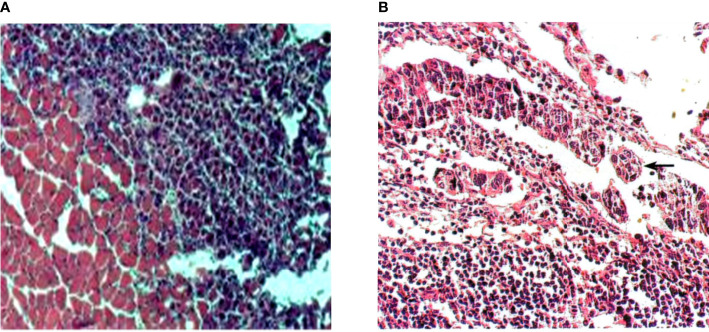
HE staining results of tissue sections. **(A)** was HE staining of adjacent tissue sections (×400); **(B)** was HE staining of tissue sections of cervical lymph node metastases (×400).

HE staining was used to analyze the pathological changes of tumor-bearing animal models. It was found that, the cancer tissue showed enlarged nuclei, darkened staining, and irregularly shaped cancer cells. The adjacent tissue mainly consisted of striated muscle, and there were oval nuclei on the edge. Observation of cervical lymph node slices revealed that, the lymphocytes in the cortex were very dense and were divided by cancer cells. The shape of the nucleus of lymphocytes was approximately round and there was no cytoplasm; while the nucleus of cancer cells was enlarged, and the cytoplasm was connected to each other into a sheet. It suggested that, the cancer cells have gradually metastasized to the lymph nodes in the neck, accompanied by cell proliferation. The specific staining results were shown in **Figure 8**.

### Detection of Naa10 and Lymphocyte Subgroups in Peripheral Blood

**Figure 9:**
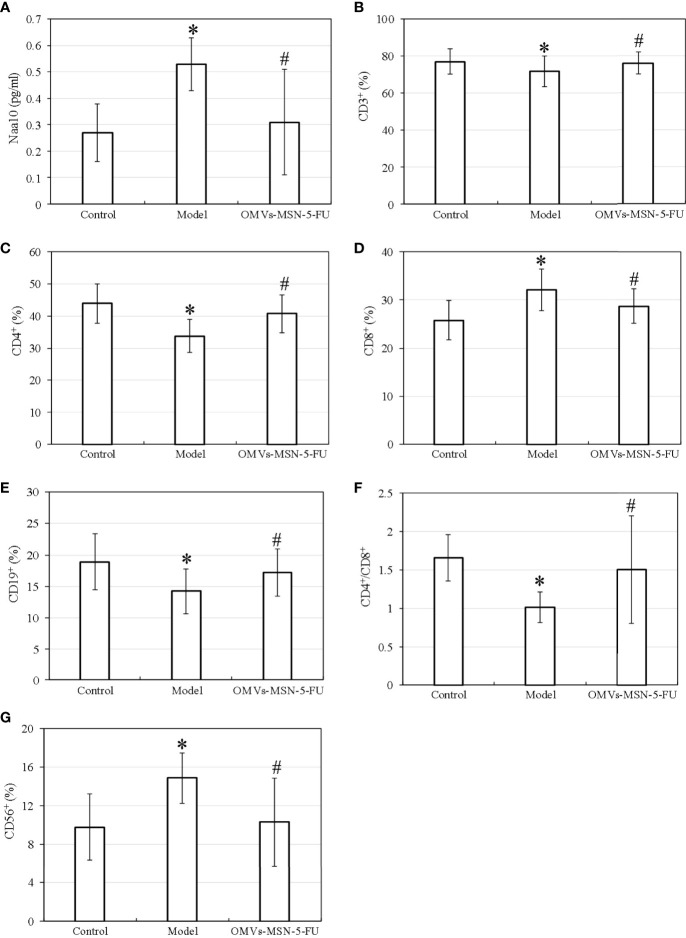
Differences in the levels of Naa10 and lymphocyte subgroups in peripheral blood. **(A)** was Naa10; **(B)** was CD3^+^ level; **(C)** was CD4^+^; **(D)** was CD8^+^; **(E)** was CD19^+^; **(F)** was CD4^+^/CD8^+^ ratio; **(G)** was CD56^+^; compared to the control group, *P<0.05; compared to the model group, ^#^ *P<*0.05.

The test results showed that, the model group showed increased levels of Naa10, CD8^+^, and CD56^+^ in peripheral blood, while decreased levels of CD3^+^, CD4^+^, CD4^+^/CD8^+^ and CD19^+^ (*P<*0.05) versus the normal control group; and that compared with the model group, the OMVs-MSN-5-FU group showed decreased levels of Naa10, CD8^+^, and CD56^+^ in the peripheral blood, while increased levels of CD3^+^, CD4^+^, CD^+^/CD8^+^ and CD19^+^ (*P<*0.05). However, there was no significant difference between the control group and the OMVs-MSN-5-FU group in the levels of Naa10 and lymphocyte subgroups (*P>*0.05). The detection results of Naa10 and lymphocyte subgroups in peripheral blood were shown in **Figure 9**.

### HE Staining of Peri-Carcinoma and Cervical Lymph Node Tissue

**Figure 10:**
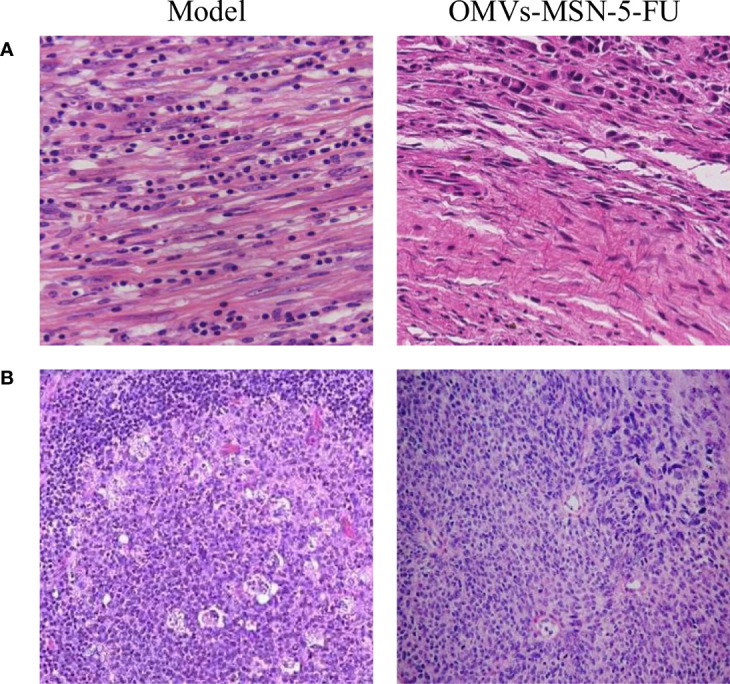
HE staining of tissue sections after treatment. **(A)** was HE staining of tissue sections adjacent to the carcinoma (×400); **(B)** was HE staining of the tissue sections of cervical lymph node metastases (×200).

The HE staining results showed that, the aligned nuclei were noted in the striated muscle of the peri-carcinoma tissue, and the injection of OMVs-MSN-5-FU reduced the number of stained cells. In the cortex of the neck lymphocytes, dense lymphocytes were clearly observed. The specific staining results were shown in **Figure 10**.

## Discussion

Patients with oral squamous cell carcinoma have a low survival rate after treatment, and metastasis may lead to poor prognosis or even death (14). There are abundant lymphatic tissues in maxillofacial region, and facial movement promotes the metastasis of oral squamous cell carcinoma to cervical lymph nodes (15). Oral squamous carcinoma has a high probability of metastasis to cervical lymph nodes. If metastasis occurs, patient survival is greatly reduced. As a thymidylate synthase inhibitor, 5-FU is often injected intravenously for the treatment of cancer patients (16). 5-FU drugs show good therapeutic effects on digestive system tumors and breast tumors. It can also be used to treat ovarian cancer, bladder cancer, and head and neck cancer (17, 18). However, the drug has a great toxic effect on bone marrow and digestive tract, so it is important to improve the therapeutic effect of the drug and reduce the toxic side effects. William (2021) (19) found that 5-FU was beneficial to improve the survival rate of colorectal cancer patients, but severe systemic toxicity (including neutropenia) occurred in 30% of patients, and 0.5-1% of patients were fatal.

In the study, Escherichia coli biofilm was used to prepare a composite nano-drug carrier system containing 5-FU drugs. OMV helps bacteria adapt to the ecological niche, and enables them to compete with others, playing a protective role (20). In this study, Escherichia coli OMV was used to wrap the MSN-5-FU drug delivery system, which was then immersed in artificial gastric juice and artificial intestinal juice to analyze the drug release rate. The results showed that, compared with MSN-5-FU, the cumulative drug release rate of OMVs-MSN-5-FU was significantly reduced. This greatly prolonged the targeted action time of the drug and improved the therapeutic effects. Finally, after co-cultured with oral squamous cell carcinoma cell lines, it was found to significantly inhibit the proliferation activity of the cells. It suggested that OMVs-MSN-5-FU had significant inhibitory effects on the proliferation activity of oral squamous cell cancer cells, and then enhanced the therapeutic effects.

Tca8113 is a type of human tongue squamous carcinoma cell line with very stable genetic traits, and has been widely used in animal experiments (21). In view of oral squamous cell carcinoma prone to neck lymphocyte metastasis, Tca8113 cells were used to prepare a tumor-bearing mouse model, and the neck lymphocyte metastasis was analyzed by making sections. The results showed that, there was obvious edema in the peri-carcinoma tissue, and the increased internal pressure caused the anchor wire connecting the endothelial cells and surrounding tissues to be pulled, which increased the pores between the lymphatic endothelial cells. In order to evaluate the therapeutic effects of OMVs-MSN-5-FU on the animal model of oral squamous cell carcinoma, the levels of Naa10 and lymphocyte subgroups in the peripheral blood were factored into. Naa10 is the only subunit that can be catalyzed in the N-acetyltransferase A complex, and it plays an important role in the cell biology process (22). Studies have shown that, Naa10 participates in the autophagy, apoptosis, and proliferation of tumor cells (23). The results of this study showed that, the level of Naa10 in the model group was significantly increased, and the injection of OMVs-MSN-5-FU could reduce the level of Naa10. Cancer cells can cause the deterioration of the disease through processes such as immune escape (24). The subgroups of peripheral blood lymphocytes were then analyzed. The results showed that, the levels of CD3^+^, CD4^+^, CD4^+^/CD8^+^ and NK cells in the peripheral blood of the model group were significantly decreased, while the levels of CD8+ and B lymphocytes were significantly increased. This indicated that the lymphocyte subgroups of model group changed significantly. After injection of OMVs-MSN-5-FU, the levels of subgroups of peripheral blood lymphocytes in the animal model almost returned to normal. This indicated that OMVs-MSN-5-FU can regulate the balance between effector T cells and helper T cells, to maintain the stability of the body’s environment, thereby improving the oral squamous cell carcinoma.

## Conclusion

To investigate the effect of OMVs-MSN-5-FU compound drugs on lymph node metastasis of oral squamous cell carcinoma, the effects of different concentrations of nano drug delivery system on the proliferation activity of KOSC-2 cl3-43 oral squamous cell carcinoma cell line were analyzed. The results showed that OMVS-MSN-5-FU compound drugs could inhibit the proliferation activity of oral squamous cell carcinoma cells, regulate the peripheral blood subsets, and inhibit the metastasis of cancer cells to cervical lymph nodes. However, some limitations should be noted. This work only analyzed the effect of the drug on the animal model of oral squamous cell carcinoma, but did not explore its internal molecular mechanism. The molecular mechanism will be further explored in the future.

## Data Availability Statement

The original contributions presented in the study are included in the article/supplementary material. Further inquiries can be directed to the corresponding author.

## Author Contributions

(I) Conception and design: JH; (II) Administrative support: JH and ZW; (III) Provision of study materials or patients: JH; (IV) Collection and assembly of data:JH and JX; (V) Data analysis and interpretation: JH, ZW,JX, (VI) Manuscript writing: All authors; (VII) Final approval of manuscript: All authors.

## Conflict of Interest

The authors declare that the research was conducted in the absence of any commercial or financial relationships that could be construed as a potential conflict of interest.

## Publisher’s Note

All claims expressed in this article are solely those of the authors and do not necessarily represent those of their affiliated organizations, or those of the publisher, the editors and the reviewers. Any product that may be evaluated in this article, or claim that may be made by its manufacturer, is not guaranteed or endorsed by the publisher.
